# Cigarette Smoke Extract, but Not Electronic Cigarette Aerosol Extract, Inhibits Monoamine Oxidase *in vitro* and Produces Greater Acute Aversive/Anhedonic Effects Than Nicotine Alone on Intracranial Self-Stimulation in Rats

**DOI:** 10.3389/fnins.2022.868088

**Published:** 2022-05-25

**Authors:** Andrew C. Harris, Peter Muelken, Aleksandra Alcheva, Irina Stepanov, Mark G. LeSage

**Affiliations:** ^1^Hennepin Healthcare Research Institute, Minneapolis, MN, United States; ^2^Department of Medicine, University of Minnesota, Minneapolis, MN, United States; ^3^Department of Psychology, University of Minnesota, Minneapolis, MN, United States; ^4^Masonic Cancer Center, University of Minnesota, Minneapolis, MN, United States

**Keywords:** nicotine, monoamine oxidase (MAO) inhibition, cigarette smoke, e-cigarette, extract, intracranial self-stimulation

## Abstract

Conventional tobacco cigarettes appear to have greater abuse liability than non-combusted products such as electronic cigarettes (ECs) and nicotine replacement therapy (NRT). This may be due to the higher levels of behaviorally active non-nicotine constituents [e.g., monoamine oxidase (MAO) inhibitors such as β-carbolines] in cigarette smoke (CS) compared to non-combusted products. To evaluate this hypothesis, the current studies compared the relative abuse liability of CS and EC aerosol extracts containing nicotine and a range of non-nicotine constituents to that of nicotine alone (NRT analog) using intracranial self-stimulation (ICSS) in rats. Effects of formulations on brain MAO activity *in vitro* and *ex vivo* were also studied to evaluate the potential role of MAO inhibition in the ICSS study. CS extract contained higher levels of several behaviorally active non-nicotine constituents (e.g., the β-carbolines norharmane and harmane) than EC extract. Nicotine alone reduced ICSS thresholds at a moderate nicotine dose, suggesting a reinforcement-enhancing effect that may promote abuse liability, and elevated ICSS thresholds at a high nicotine dose, suggesting an aversive/anhedonic effect that may limit abuse liability. CS extract elevated ICSS thresholds to a greater degree than nicotine alone at high nicotine doses. Effects of EC extract on ICSS did not differ from those of nicotine alone. Finally, CS extract significantly inhibited MAO-A and MAO-B activity *in vitro*, whereas EC extract and nicotine alone did not. None of the formulations inhibited MAO measured *ex vivo*. These findings indicate greater acute aversive/anhedonic effects for CS extract compared to nicotine alone, suggesting lower abuse liability. Although confirmation of our findings using other dosing regimens, preclinical addiction models, and tobacco product extracts is needed, these findings suggest that the centrally-mediated effects of MAO inhibitors and other non-nicotine constituents may not account for the greater abuse liability of cigarettes compared to non-combusted products. Nonetheless, identifying the specific constituent(s) mediating the effects of CS extracts in this study could help clarify mechanisms mediating tobacco addiction and inform FDA product standards.

## Introduction

Several lines of evidence suggest that combusted tobacco cigarettes have greater abuse potential than non-combusted, alternative nicotine delivery systems (ANDS) such as electronic cigarettes (ECs), smokeless tobacco, and nicotine replacement therapy (NRT). For example, combusted cigarettes are associated with higher rates of use among adults, greater reinforcement efficacy, and higher levels of dependence compared to ANDS (e.g., Etter and Eissenberg, 2015; Stein et al., 2017; Stiles et al., 2017; National Academies of Sciences, Engineering, and Medicine, 2018; Cornelius et al., 2020; Shiffman and Sembower, 2020). Understanding the product characteristics (e.g., constituents) that contribute to the differential abuse liability of cigarettes versus ANDS could inform FDA regulation of tobacco product design features and constituent levels. Such work could also provide insights into the basic behavioral and neurobiological mechanisms contributing to tobacco addiction and lead to the development of more effective treatments.

Nicotine is the primary addictive constituent in tobacco products, and the variance in nicotine content/yield between products could contribute to differences in abuse liability between cigarettes and ANDS. However, certain ANDS (e.g., newer-generation ECs) can produce levels of nicotine exposure similar to or even higher than combustible cigarettes (e.g., Lopez et al., 2016; Ramoa et al., 2016; Spindle et al., 2017; Hajek et al., 2020). Furthermore, several findings indicate that factors other than nicotine contribute to tobacco product abuse liability. For example, nicotine alone has limited abuse potential in humans and animals (Rose et al., 2000; Manzardo et al., 2002; Le Foll and Goldberg, 2009), and switching smokers to very low nicotine content cigarettes results in only a limited reduction in cigarettes smoked per day (Donny et al., 2007, 2015; Benowitz et al., 2012).

While there are numerous factors other than nicotine content/yield that could contribute to the greater abuse liability of cigarettes versus ANDs (e.g., sensory effects, marketing, etc.), cigarette smoke (CS) contains considerably higher levels of several behaviorally active non-nicotine constituents including monoamine oxidase (MAO) inhibitors (e.g., the β-carbolines harmane and norharmane, also called harman and norharman), minor tobacco alkaloids (e.g., nornicotine, anabasine), and volatile organic compounds (VOCs) (e.g., acetaldehyde, toluene) (e.g., Margham et al., 2016; National Academies of Sciences, Engineering, and Medicine, 2018; Belushkin et al., 2020). These compounds can mimic or enhance nicotine’s addiction-related effects in preclinical models, or may have abuse liability themselves (for review, see Hoffman and Evans, 2013; LeSage et al., 2018). The greater abuse liability of cigarettes versus ANDs may therefore reflect the higher levels of MAO inhibitors and other behaviorally active non-nicotine constituents in CS.

A useful preclinical approach for understanding determinants of tobacco abuse involves administration of extracts derived directly from tobacco products that contain nicotine and a wide range of non-nicotine constituents (e.g., Brennan et al., 2014, 2015; Costello et al., 2014). In contrast to traditional animal models of tobacco addiction that involve administration of nicotine alone or other isolated tobacco constituents, the use of extracts allows for evaluation of the aggregate effects of exposure to a range of tobacco constituents as occurs during actual product use. Although interactions can also be evaluated using inhalational models (e.g., Brennan et al., 2014; Chellian et al., 2021), parenteral (e.g., i.v., s.c.) administration of extracts allows the direct study of addiction-related central nervous system (CNS) effects of tobacco constituents largely independent of their peripheral (e.g., sensory) effects, avoidance of stressors associated with inhalational exposure, and more precise dosing (e.g., Brennan et al., 2014). Several studies have found greater addiction-related behavioral or neurobiological effects of CS extracts compared to nicotine alone (e.g., Brennan et al., 2014; Costello et al., 2014; Marusich et al., 2019), which may reflect the effects of behaviorally active non-nicotine constituents in the extracts.

While previous studies evaluating the addiction-related effects of CS extracts have been extremely valuable, they also have several limitations. First, nearly all of these studies have used extracts of only the particulate phase of CS, which excludes or limits levels of addiction-relevant constituents (e.g., VOCs such as acetaldehyde and toluene) that occur in the gas phase of CS. Second, several of these studies have used saline as a solvent for extract preparation (e.g., Costello et al., 2014; Gellner et al., 2016; Marusich et al., 2019), which limits or prevents extraction of constituents that have poor water solubility (e.g., β-carbolines). Third, chemical analyses of non-nicotine constituents in CS extracts have typically been limited to measuring 2–3 compounds (Brennan et al., 2013a,^2015^; Lee et al., 2016) or not conducted at all (Brennan et al., 2013b; Costello et al., 2014; Gellner et al., 2016; Cross et al., 2020; Levin et al., 2021), preventing identification of the non-nicotine constituents contributing to any unique effects of the extracts. Finally, comparisons of CS extracts and extracts of other classes of tobacco products (e.g., ECs) have rarely been conducted in the same study (Marusich et al., 2019), preventing characterization of their relative abuse liability. A goal of the present study was to address these limitations.

Abuse liability in this study was measured using intracranial self-stimulation (ICSS), which provides a sensitive index of the function of brain reinforcement systems. Low to moderate doses of nicotine reduce the lowest (threshold) stimulation intensity that maintains operant responding for ICSS, reflecting nicotine’s ability to enhance the reinforcing effects of other stimuli (Kornetsky et al., 1979; Huston-Lyons and Kornetsky, 1992; Rupprecht et al., 2015; Kenny et al., 2018). At high doses, nicotine attenuates the reinforcing effects of the brain stimulation and increases ICSS thresholds, providing a putative marker of nicotine’s acute aversive/anhedonic effects (Kenny et al., 2003; Spiller et al., 2009; Fowler et al., 2011). Nicotine’s reinforcement-enhancing and aversive effects may both influence tobacco abuse liability (Sellings et al., 2008; Fowler and Kenny, 2014; Rupprecht et al., 2015). For example, because nicotine’s aversive effects can limit its intake (e.g., Verendeev and Riley, 2013; Fowler and Kenny, 2014; Wills et al., 2022), reductions in nicotine aversion could increase tobacco use. The acute effects of a drug on ICSS are generally predictive of its abuse liability in other preclinical models (e.g., i.v. self-administration) and in humans (Wise, 1996; Negus and Miller, 2014). Supporting the utility and sensitivity of ICSS for tobacco constituent evaluation, we have used this methodology to evaluate the relative abuse liability of smokeless tobacco extracts (Harris et al., 2012, 2015b), EC liquids (LeSage et al., 2016; Harris et al., 2017, 2018b), and isolated non-nicotine constituents [i.e., β-carbolines, minor alkaloids, and the EC solvent propylene glycol (PG) (Harris et al., 2015a, 2018a, 2020)]. However, effects of CS or EC aerosol extracts on ICSS have not been evaluated.

The primary purpose of this study was to compare the acute effects of nicotine alone and CS extract or EC aerosol extract on ICSS. Extracts were prepared from either Marlboro Gold cigarettes or Vuse Solo Original (tobacco-flavor) ECs, two of the most popular brands of their respective classes. Vuse Solo Original is also of interest because it recently became the first EC approved for marketing by the FDA under a Premarket Tobacco Product Application (PMTA). Extracts were prepared using an approach that captures water-soluble and water-insoluble constituents from both the particulate and gas phase of CS and EC aerosol. To evaluate potential constituents that could account for any behavioral effects of extracts and to ensure consistency between batches of extracts, we evaluated levels of a range of behaviorally relevant non-nicotine constituents including MAO inhibitors (harmane and norharmane), minor alkaloids (e.g., nornicotine, anabasine), carbonyls (e.g., acetaldehyde, acetone), and glycols (e.g., PG). We also measured several constituents that are known toxicants but whose behavioral effects are unknown (e.g., crotonaldehyde, ethylene glycol). Effects of extracts and nicotine alone on brain MAO activity *in vitro* and *in vivo* were also studied to evaluate the potential role of MAO inhibition in our findings. MAO inhibition was studied because other CS extracts and β-carbolines present in cigarette smoke (e.g., harmane, norharmane) inhibit MAO activity *in vitro* (e.g., Herraiz and Chaparro, 2005; Lewis et al., 2012; Costello et al., 2014). This mechanism is also of interest because brain levels of the MAO-A and MAO-B isozymes are inhibited in smokers, an effect that may contribute to tobacco addiction (Fowler et al., 1996; Berlin and Anthenelli, 2001).

## Materials and Methods

### Animals

Experimentally-naive male and female Sprague Dawley rats (Envigo, Indianapolis, IN, United States) weighing 250–299 g (males) or 200–250 g (females) at arrival were housed individually in a temperature- and humidity-controlled colony room with unlimited access to water. Rats were housed under a reversed 12-h light/dark cycle and tested during the dark (active) phase. Beginning one week after arrival, rats were food-restricted to ≈18 g/day (males) or ≈16 g/day (females) rat chow to facilitate operant performance and avoid detrimental effects of long-term *ad libitum* feeding on health. Protocols were approved by the Institutional Animal Care and Use Committee of the Hennepin Healthcare Research Institute in accordance with the NIH Guide for the Care and Use of Laboratory Animals and the Guidelines for the Care and Use of Mammals in Neuroscience and Behavioral Research.

### Drug Preparation

Nicotine-alone solutions consisted of (-)-nicotine (Sigma Chemical Co., St. Louis, MO, United States) dissolved in sterile saline. CS extracts were prepared from Marlboro Gold cigarettes purchased in the Minneapolis area. Cigarettes were conditioned for 48 h in an environmental chamber at 22°C and 60% relative humidity. Cigarettes were then smoked on a Borgwaldt LX1 linear single port smoking machine under the Canadian Intense smoking regimen (55-ml puff volume, 2-s puff duration, 30-s puff interval, and 100% blockade of filter ventilation holes). Total particulate matter (TPM) was collected on Cambridge filter pads, while gas phase constituents were trapped in an impinger containing 10 ml 50:50 saline/ETOH maintained at −23°C. After a total of 40 cigarettes were smoked (five cigarettes per filter pad), filter pads were extracted in 10 ml of a 50:50 saline/ETOH mixture. Use of ETOH for extraction captures a range of constituents, including those that are volatile and/or non-water soluble. ETOH has also been used as a solvent in numerous preclinical studies evaluating addiction-related effects of extracts. (e.g., Brennan et al., 2013a,b, 2015). The TPM fraction was cooled to -20°C and combined with the gas phase constituents to make the final CS extract (volume ≈ 17.5 ml, see below for constituent levels in undiluted extracts).

Electronic cigarette extracts were prepared in a similar manner. EC aerosol was generated using a Vuse Solo device containing Vuse Original (tobacco-flavored) EC liquid (4.8% nicotine concentration) in cartridges (device and cartridges purchased from vusevapor.com). Puffing parameters were in the range of those observed in experienced EC users (Kosmider et al., 2018). A total of 800 puffs (100 puffs per filter pad) were puffed (100-ml puff volume, 4-s puff duration and 40-s interval between puffs) and final EC extracts were prepared as described above.

The nicotine concentration was determined for CS and EC extract as described below. Extracts were then diluted to the nicotine concentrations required for the current studies. Thus, the concentration of all constituents in extract decreased in proportion to nicotine. Because ETOH was used as a solvent for extract preparation, both CS and EC extracts contained ≈ 0.75% – 32% ETOH (delivering ≈ 5.9 – 252.5 mg/kg ETOH) after dilution depending on nicotine dose (0.03 – 1.25 mg/kg). To control for potential interactions between nicotine and ETOH, the same respective ETOH concentrations present in the extract doses were added to the nicotine alone doses (e.g., 32% ETOH was added to the 1.25 mg/kg nicotine alone dose, 25% ETOH was added to the 1.0 mg/kg nicotine alone dose, etc.). All extract solutions were stored and administered in Hamilton gas-tight syringes (Hamilton Company, Reno NV) to minimize loss of volatile constituents. The pH of all solutions was adjusted to 7.4 using dilute NaOH. Nicotine doses are expressed as the base. All injections were administered s.c. in a volume of 1 ml/kg.

### Chemical Analyses of Extracts

#### Nicotine and Minor Alkaloids

Nicotine and minor alkaloids samples were analyzed with modifications of a previously described method (Jain et al., 2019). Briefly, samples were prepared by serial dilution of the CS and EC extract with 10 mM ammonium acetate, and addition of [D_3_]nicotine, [D_4_]nornicotine, [D_4_]anabasine and [D_4_]anatabine as internal standard. Samples were then analyzed by liquid chromatography-tandem mass spectrometry (LC-MS/MS) on a Hypercarb column (Thermo Scientific), using 10 mM ammonium acetate (with 0.01% formic acid) and methanol as mobile phase.

#### β-Carbolines

For analysis of harmane and norharmane, CS and EC extracts (50 μl) were mixed with ^13^C_2_^15^N-harman and D_7_-norharman internal standards and diluted to 5 ml with water. The mixture was subjected to purification by solid supported liquid-liquid extraction using ChemElut cartridges (Agilent, Santa Clara, CA) and extracted using 8 ml methylene chloride twice. The samples were dried in SpeedVac for 1.5 h and reconstituted in 1 ml of deionized water. Samples were then analyzed by LC-MS/MS on a Zorbax SB C18 (Agilent, Santa Clara, CA) column, using water (with 0.1% trifluoroacetic acid) and acetonitrile (with 0.1% trifluoroacetic acid) as mobile phase. The mass spectrometer with electrospray ionization source was set in the positive ion mode with selective reaction monitoring (SRM) monitoring *m/z* 169→115 and *m/z* 183→115 for norharmane and harmane, respectively.

#### Carbonyls

Cigarette smoke and EC extracts were analyzed for eight carbonyl compounds with modifications of a previously described method (Jain et al., 2019). Briefly, CS and EC extracts (100 μl) were derivatized with 2,4-dinitrophenylhydrazine (DNPH) for 20 min. The derivatization reaction was stopped with 1% Trizma base and samples were analyzed by high-performance liquid chromatography (HPLC-UV) on a Phenomenex C18(2) 250 mm × 4.6 mm column, using 30% acetonitrile/10% tetrahydrofuran/1% isopropanol/59% water as mobile phase A and 65% acetonitrile/1% tetrahydrofuran/1% isopropanol/33% water as mobile phase B, with the UV detector set at 365 nm.

#### Glycols

Glycol concentrations were measured by gas chromatography (GC) with flame ionization detection (FID) on an Agilent 6890 GC with ChemStation, V10.1. 1,3-propanediol (trimethylene glycol) was utilized as an internal standard. Levels greater than the limit of linearity (100 mg/dl) are diluted.

### Experiment 2: Effects of Cigarette Smoke Extract, Electronic Cigarette Extract, and Nicotine Alone on Intracranial Self-Stimulation

#### Intracranial Self-Stimulation

Surgery, apparatus, and training procedure used here are described in detail elsewhere (e.g., Harris et al., 2010, 2011; Swain et al., 2020). Briefly, animals were anesthetized with i.m. ketamine (75 mg/kg)/dexmedetomidine (0.025 mg) and implanted with a bipolar stainless steel electrode in the medial forebrain bundle at the level of the lateral hypothalamus. Rats were later trained to respond for electrical brain stimulation using a modified version of the Kornetsky and Esposito (1979) discrete-trial current-threshold procedure (Markou and Koob, 1992). Each session lasted ≈ 45–60 min and provided two dependent variables: ICSS thresholds (a measure of the function of brain reinforcement systems) and response latencies [a measure of non-specific (e.g., motor) effects].

### Experiment 2a: Effects of Nicotine Alone and Cigarette Smoke Extract on Intracranial Self-Stimulation

Rats (*N* = 21, 10–11/sex) were tested in daily ICSS sessions conducted Mon-Fri until ICSS thresholds were stable (i.e., <10% coefficient of variation over a 5-day period and no apparent trend). To habituate animals to the injection procedure, saline was administered 10 min prior to ICSS testing twice per week (Tuesdays and Fridays) for at least one session and until thresholds were stable. Effects of 10-min pretreatment with nicotine alone (half of the rats of each sex) or CS extract (the other half) were subsequently determined at nicotine doses of 0 (i.e., saline alone), 0.03, 0.125, 0.25, 1.0, or 1.25 mg/kg. These nicotine doses reduce or increase ICSS thresholds when administered acutely (Huston-Lyons and Kornetsky, 1992; Bauco and Wise, 1994; Harrison et al., 2002; Spiller et al., 2009; Harris et al., 2012). Because CS extract and nicotine alone doses contained ≈0.75–32% ETOH depending on the dilution (see above), rats also received an injection of 32% ETOH alone during dose-response testing to evaluate its potential effects on ICSS in the absence of nicotine. Injections were typically administered on Tuesdays and Fridays, provided that ICSS thresholds were within baseline range on intervening days, and doses were administered in a counterbalanced order. Following completion of dose-response testing, animals were tested for ICSS under drug-free conditions for at least 2 weeks and until ICSS thresholds were stable. All rats then underwent the same procedure as described above except that formulation (i.e., nicotine alone vs. CS extract) was crossed-over within each subject.

### Experiment 2b: Effects of Nicotine Alone and Electronic Cigarette Extract on ICSS

A separate group of 20 rats (10 rats/sex) was tested as described in Experiment 2a, except that rats were administered EC aerosol extract rather than CS extract.

### Experiment 3: Effects of Cigarette Smoke Extract, Electronic Cigarette Extract, and Nicotine Alone on Monoamine Oxidase Activity

#### Monoamine Oxidase Assay

To prepare brain homogenate, one half hemisphere of rat brain was homogenized with 4 ml sodium phosphate buffer (50 mM, pH 7.4) and centrifuged at ∼600 × *g* for 10 min at 4°C. The supernatant was then diluted with buffer to a final tissue concentration of 20 mg/ml (protein concentration ≈600 μg/ml). MAO activity was subsequently measured using the Amplex Red Monoamine Oxidase Assay (ThermoFisher Scientific, Waltham, MA, United States). This assay involves the detection of H_2_O_2_, a marker of MAO activity, in a horseradish peroxidase (HRP)-coupled reaction using a reagent (Amplex Red, or 10-acetyl-3,7dihydroxyphenoxazine) that is sensitive to H_2_O_2_. The assay was conducted according to the manufacturer’s instructions. Briefly, 200 μl of each sample was combined with 200 μl reaction buffer and 20 μl of inhibitor (protein concentration following dilution ≈275 μg/ml). After a 30-min pre-incubation period at room temperature, 100 μl of this solution was plated in triplicate on a 96-well microplate with 100 μl of a working solution containing 400 μM Amplex Red reagent, 2 mM tyramine (a substrate for MAO-A and MAO-B), and 2.0 U/ml HRP in reaction buffer (protein concentration of final reaction ≈138 μg/ml). 100 μl of the working solution was also added to either 100 μl of 10 μM H_2_O_2_ in buffer as a positive control, or to 100 μl of buffer alone as a negative control. The microplate was then allowed to incubate for 60 min at room temperature while protected from light. Levels of resorufin (fluorescence emission maxima = 585 nm), a fluorescent reaction product that is proportional to H_2_O_2_ generation, were subsequently read using a fluorescence microplate reader (BioTek Cytation, Biotek Instruments, Inc, Winooski, VT, United States) using excitation at 545 ± 15 nm and fluorescence detection at 590 ± 10 nm.

### Experiment 3a: Effects of Extracts and Nicotine Alone on MAO Inhibition *in vitro*

To validate the MAO assay, the procedure described above was conducted using a range of concentrations of the non-selective MAO inhibitor tranylcypromine (TCP, Sigma Chemical Co.). To compare the effects of extracts and nicotine alone, this procedure was repeated except that either saline or a range of concentrations of CS extract, EC extract, or nicotine alone was used as the inhibitor. Because nicotine alone and extracts contained ≈ 0.02–32% ETOH depending on the nicotine concentration (0.625–1,250 μg/ml), a 32% ETOH condition was included to evaluate its potential effects in the assay in the absence of nicotine. Background activity was measured in the presence of 20 μl TCP at a concentration (0.5 mg/ml) that completely inhibited MAO in the initial validation study. Following demonstration that CS extract inhibited total MAO activity, a follow-up study was conducted using only CS extract to evaluate the relative contributions of MAO-A and MAO-B to this effect. Assays were conducted as described above, except that MAO-B activity was measured by addition of 20 μl of the selective MAO-A inhibitor clorgyline (5 μM). MAO-A activity was determined as the difference between total MAO activity and MAO-B activity.

### Experiment 3b: Effects of Extracts and Nicotine Alone on Monoamine Oxidase Inhibition *ex vivo*

Rats (*n* = 6/group, 3 rats/sex) were injected s.c. with either saline or CS extract, EC extract, or nicotine alone at a nicotine dose of 1.25 mg/kg (i.e., the highest nicotine dose used in the behavioral experiment). An additional positive control group (*n* = 6, 4 females, 2 males) was injected with TCP at a dose (3.0 mg/kg, i.p.) that fully inhibits MAO measured *ex vivo* (e.g., Villegier et al., 2007a,b). One hour later, rats were anesthetized using isoflurane and rapidly decapitated. Brain homogenates were then prepared and assayed for MAO activity using the same general procedure described above. Protein concentration (BCA assay, Sigma Chemical St. Louis, MO, United States) was also measured in triplicate 10 μl aliquots of the supernatant fluid so that MAO activity values could be expressed per milligram protein.

### Statistical Analyses

In Experiment 2a, baseline ICSS thresholds (in μA) and response latencies (in s) were defined as the mean during the last five sessions prior to dose-response testing. These baseline data were compared between nicotine alone and CS extract dose-response assessments using separate two-factor ANOVAs with sex as a between-subject factor and formulation (i.e., nicotine alone versus CS extract) as a within-subject factor. All ICSS data were subsequently expressed as % baseline. Because paired-samples *t*-tests comparing the saline alone and 32% ETOH alone conditions showed no significant difference, these conditions were combined into a single vehicle (negative control) condition. ICSS threshold and latency values during test sessions were subsequently compared using separate three-factor ANOVAs with sex as a between-subject factor and formulation and nicotine dose as within-subject factors. Because there was an effect of formulation and a formulation × nicotine dose interaction, but no effects of sex or interactions related to this variable, data were collapsed across sex and compared between formulations using Holm-Sidak *post hoc* tests at each nicotine dose. Data within each formulation were also analyzed using Dunnett’s *post hoc* tests comparing each nicotine dose to vehicle. Degrees of freedom for all ANOVAs were adjusted using the Greenhouse-Geisser correction to account for possible violations of sphericity. In the few cases in which a rat failed to respond for any ICSS current intensity, we assigned ICSS threshold and latency values based on those obtained in the animal achieving the highest ICSS threshold (see Markou and Koob, 1991; Harris et al., 2015a, 2018a; Swain et al., 2020). Data from Experiment 2b (nicotine alone versus EC extract) were analyzed in the same manner.

In Experiment 3a, concentration-response curves were produced using a standard inhibition model (four parameter, variable slope fit of log concentration vs. fluorescence). The amount causing half-maximal inhibition (IC50) for each formulation is expressed in μg/ml. Curve fitting was performed using Prism (Graphpad software, San Diego, CA, United States). In Experiment 3b, total MAO activity (expressed as % control) was compared between formulations using a Brown-Forsythe ANOVA followed by Dunnett T3 *post hoc* tests (to account for unequal variances) comparing each formulation to vehicle. Sex was not included as a factor in Experiment 3b due to the low number of rats of each sex that were tested. In all experiments, *p*-values < 0.05 were considered statistically significant.

## Results

### Experiment 1: Constituent Levels in Extracts

**FIGURE 1:**
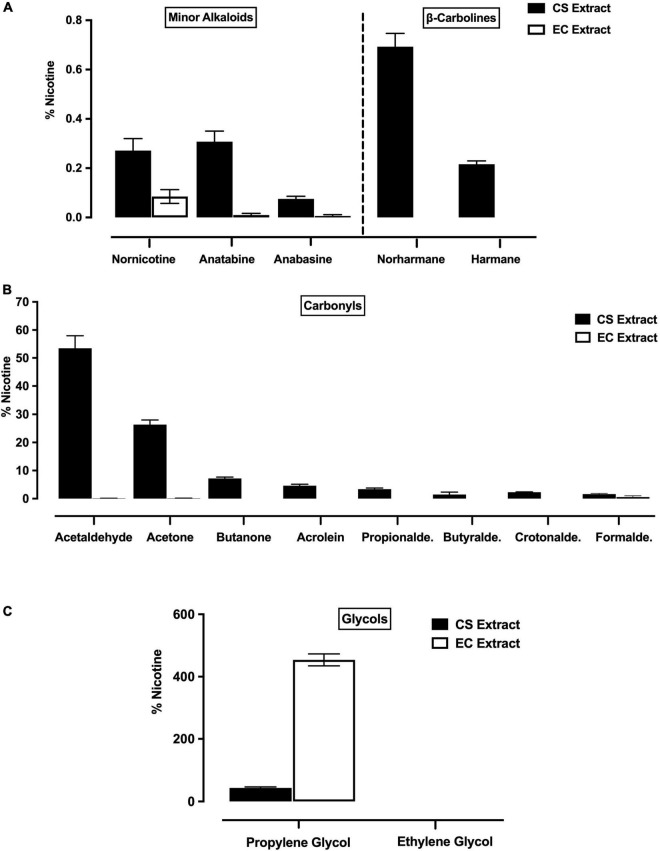
Levels (expressed as % of nicotine, mean ± SEM) of minor alkaloids (**A**, left panel), β-carbolines (**A**, right panel), carbonyls **(B)** and glycols **(C)** in CS extract and EC extract. Minor alkaloids and carbonyls were measured in all eight representative batches of CS and EC extract, whereas β-carbolines and glycols were measured in three of these batches.

Nicotine levels (mean mg/ml ± SEM across eight representative, undiluted batches of each extract) were similar in Marlboro Gold CS extract (3.03 ± 0.14 mg/ml) and Vuse Solo Original EC extract (2.98 ± 0.10 mg/ml). When expressed as % of nicotine, levels of minor alkaloids, β-carbolines, and carbonyls were higher in undiluted CS extract compared to undiluted EC extract (Figures 1A,B). In contrast, levels of PG were higher in EC extract than in CS extract (Figure 1C). Ethylene glycol was not detected in either CS or EC extract (Figure 1C).

### Experiment 2: Effects of Nicotine Alone and Cigarette Smoke Extract or Electronic Cigarette Extract on Intracranial Self-Stimulation

#### Experiment 2a: Effects of Nicotine Alone and Cigarette Smoke Extract on Intracranial Self-Stimulation

All data from one male and three females were excluded due to loss of ICSS headcap or loss of stable ICSS thresholds. The remaining 17 rats (10 males, 7 females) required 39.4 ± 5.3 (mean ± SEM) total ICSS sessions to achieve stable ICSS thresholds. Evaluation of baseline ICSS thresholds and response latencies in these rats indicated no effect of sex, formulation, or sex × formulation interaction for either baseline ICSS thresholds or baseline ICSS latencies (Table 1).

**TABLE 1:** Mean (±SEM) baseline ICSS thresholds (in μA) and response latencies (in s) in males and females during nicotine alone and CS or EC extract testing in Experiments 2a and 2b.

	Male		Female	
	Threshold (μA)	Latency (s)	Threshold (μA)	Latency (s)
**Experiment 2a**				
Nicotine alone	84.9 ± 5.8	2.6 ± 0.2	92.8 ± 22.8	2.9 ± 0.1
CS extract	90.6 ± 6.2	2.7 ± 0.2	88.8 ± 19.7	2.8 ± 0.2
**Experiment 2b**				
Nicotine alone	92.8 ± 11.2	2.9 ± 0.2	99.8 ± 12.5	2.5 ± 0.2
EC extract	86.6 ± 12.9	2.8 ± 0.2	97.5 ± 9.6	2.5 ± 0.2

**FIGURE 2:**
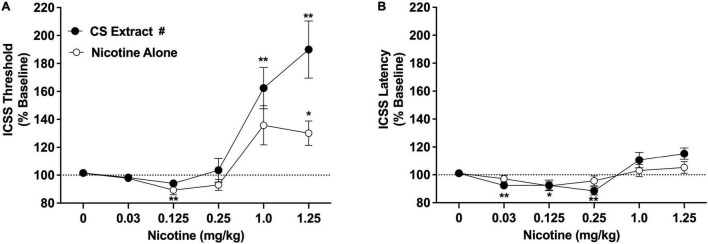
Intracranial self-stimulation (ICSS) thresholds **(A)** and response latencies **(B)** (expressed as percent of baseline, mean ± SEM) following injection of nicotine alone or CS extract (0–1.25 mg/kg) in Experiment 2a. ^#^Significant main effect of formulation, *p* < 0.05. *,** Significantly different from vehicle (0 mg/kg) for that formulation, *p* < 0.05 or 0.01.

Intracranial self-stimulation thresholds (expressed as % baseline) in the saline versus 32% ETOH conditions did not differ during either the nicotine alone dose-response function (100.2 ± 1.6% vs. 102.2 ± 2.3% collapsed across sexes, which did not differ) or the CS extract dose-response function (101.5 ± 2.8% vs. 100.7 ± 2.7% collapsed across sexes, which did not differ), and were therefore collapsed into a single negative control condition for each dose-response assessment. There were statistically significant main effects of nicotine dose [*F*(2.2, 34.8) = 25.3, *p* < 0.0001] and formulation [*F*(1,16) = 5.9, *p* < 0.05], and a significant nicotine dose × formulation interaction [*F*(2.3,34.9) = 3.6, *p* < 0.05]. There was no main effect of sex and no interactions related to this variable. Comparisons between formulations collapsed across sex indicated that ICSS thresholds did not differ between CS extract and nicotine alone at any dose (Figure 2A). For the nicotine alone condition, ICSS thresholds were significantly reduced compared to vehicle at 0.125 mg/kg (Dunnett q = 4.7, *p* < 0.01) and elevated compared to vehicle at 1.25 mg/kg (q = 3.2, *p* < 0.05) (Figure 2A). For CS extract, ICSS thresholds were not significantly reduced compared to vehicle at any nicotine dose, and were elevated compared to vehicle at both the 1.0 mg/kg and 1.25 mg/kg doses (q = 4.2 and 4.4, respectively, *p* < 0.01; Figure 2A).

Intracranial self-stimulation latencies in the saline and 32% ETOH conditions did not differ during either the nicotine alone dose-response function or the CS extract dose-response function (data not shown) and were therefore collapsed into a single vehicle condition for each dose-response assessment. There was no effect of formulation on ICSS latencies, but there were significant effects of nicotine dose [*F*(2.0, 32.3) = 8.36, *p* < 0.0001] and a significant nicotine dose × formulation interaction [*F*(3.9,58.1) = 3.3, *p* < 0.05]. There was no effect of sex or interactions related to this variable. Analysis of data collapsed across sex indicated that ICSS latencies did not differ between CS extract and nicotine alone at any nicotine dose (Figure 2B). For nicotine alone, ICSS latencies were reduced compared to vehicle at the 0.125 mg/kg dose (q = 3.2, *p* < 0.05). For CS extract, ICSS latencies were reduced compared to vehicle at the 0.03 and 0.25 mg/kg doses (q = 3.6 and 4.2, respectively, *p* < 0.01; Figure 2B). No other significant differences were observed (Figure 2B).

#### Experiment 2b: Effects of Nicotine Alone and Electronic Cigarette Extract on Intracranial Self-Stimulation

Data from two males and three females were excluded due to the same issues described in Experiment 2a. The remaining 15 rats (9 males, 6 females) achieved stable ICSS thresholds following 42.5 ± 4.7 (mean ± SEM) total ICSS sessions. There was no effect of sex, formulation, or interaction on baseline ICSS thresholds or baseline ICSS latencies in these animals (Table 1).

**FIGURE 3:**
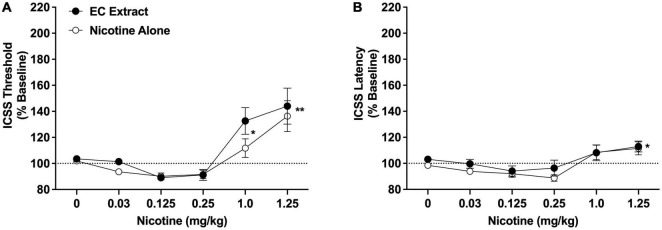
Intracranial self-stimulation (ICSS) thresholds **(A)** and response latencies **(B)** (expressed as percent of baseline, mean ± SEM) following injection of nicotine alone or EC extract (0–1.25 mg/kg) in Experiment 2b. *,**Significantly different from vehicle (0 mg/kg), collapsed across both formulations, *p* < 0.05 or 0.01.

Intracranial self-stimulation thresholds in the saline versus 32% ETOH conditions did not differ during either the nicotine alone dose-response function (102.9 ± 1.7% vs. 101.4 ± 1.5% collapsed across sexes, which did not differ) or the EC extract dose-response function (103.4 ± 2.1% vs. 103.6 ± 1.3% collapsed across sexes, which did not differ), and were therefore collapsed into a single vehicle condition for each dose-response assessment. There was a significant main effect of nicotine dose on ICSS thresholds [*F*(5, 65) = 18.2, *p* < 0.0001], but no effect of formulation, sex, or interactions related to these variables. Comparison of data collapsed across sex and formulation indicated that thresholds were elevated compared to vehicle at the 1.0 mg/kg (q = 2.9, *p* < 0.05) and 1.25 mg/kg (q = 5.6, *p* < 0.01) nicotine doses (Figure 3A). The apparent reductions in ICSS thresholds at the 0.125 and 0.25 mg/mg doses compared to vehicle (see Figure 3A) were not significant (*p* > 0.20).

Intracranial self-stimulation latencies in the saline and 32% ETOH conditions did not differ during either the nicotine alone or EC extract dose-response function (data not shown) and were collapsed into a single vehicle condition for each dose-response assessment. There was a significant main effect of nicotine dose on ICSS latencies [*F*(5, 65) = 8.5, *p* < 0.0001], but no effect of formulation, sex, or interactions related to these variables. Comparison of data collapsed across sex and formulation indicated that latencies were elevated compared to vehicle at the 1.25 mg/kg nicotine dose (q = 2.8, *p* < 0.05; Figure 3B). No other nicotine dose differed significantly from vehicle.

### Experiment 3: Effects of Cigarette Smoke Extract, Electronic Cigarette Extract, and Nicotine Alone on Monoamine Oxidase Activity

#### Experiment 3a: Effects of Cigarette Smoke Extract, Electronic Cigarette Extract, and Nicotine Alone on Monoamine Oxidase Activity *in vitro*

**FIGURE 4:**
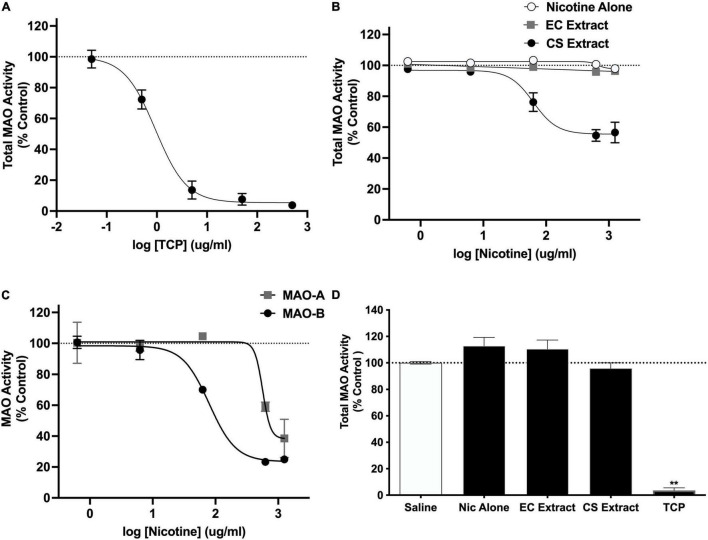
Total MAO activity (expressed as % control) in brain homogenate following *in vitro* application of various concentrations of **(A)** TCP or **(B)** nicotine alone, CS Extract, or EC extract. Panel **(C)** shows MAO-A and MAO-B activity (% control) following *in vitro* application of various concentrations of CS extract. Data in panels **(A–C)** represent the mean ± SEM across 2–5 replications. Panel **(D)** shows total MAO activity (% control) in rat brain measured *ex vivo* following s.c. injection of saline or 1.25 mg/kg nicotine alone, CS extract, or EC extract, or i.p. injection of 3.0 mg/kg TCP (positive control). **Different from saline, *p* < 0.0001. Absolute fluorescence values (relative fluorescence units, mean ± SEM) for controls for panels **(A–D)** were 397,509 ± 21,044, 349,888 ± 40,700, 365,544 ± 68,497, and 357,593 ± 16,850, respectively.

In the initial validation study, TCP produced a concentration-dependent inhibition of total MAO activity (IC50 = 0.94 μg/ml; Figure 4A). Total MAO activity did not differ between the saline and 32% ETOH conditions in the subsequent two studies evaluating effects of extracts (mean % difference ± SEM = 2.6 ± 1.8% and 1.3 ± 9.2%, respectively). Data from these conditions were therefore combined into a single negative control condition in both studies. CS extract produced a concentration-dependent inhibition of total MAO activity (IC50 = 62.7 μg/ml; Figure 4B). In contrast, nicotine alone and EC extract did not affect MAO activity at any nicotine concentration. CS extract inhibited both the MAO-A and MAO-B isoforms (Figure 4C), although its potency for inhibiting MAO-B was greater than that for MAO-A (IC50 for MAO-B and MAO-A = 79.5 and 565.0 μg/ml, respectively).

#### Experiment 3b: Effects of Cigarette Smoke Extract, Electronic Cigarette Extract, and Nicotine Alone on Monoamine Oxidase Activity *ex vivo*

There was a significant effect of formulation on total MAO activity [*F*(4,13.7) = 100.8, *p* < 0.0001], although only the TCP (positive control) condition differed from the negative control condition (t = 80.2, *p* < 0.0001; Figure 4D).

## Discussion

The current studies compared the relative abuse liability of Marlboro Gold CS extract or Vuse Solo Original EC aerosol extract to that of nicotine alone (NRT analog) using ICSS in rats. In summary, chemical analyses indicated that CS extract contained higher levels of several behaviorally active non-nicotine constituents [e.g., β-carbolines (MAO inhibitors), minor alkaloids] than EC extract. Nicotine alone reduced ICSS thresholds at a moderate nicotine dose, indicating a reinforcement-enhancing effect, and elevated ICSS thresholds at a high nicotine dose, suggesting an aversive/anhedonic effect. The significant main effect of formulation and formulation × dose interaction in Experiment 2a indicate that CS extract elevated ICSS thresholds compared to nicotine alone at high nicotine doses (1.0 and 1.25 mg/kg, see Figure 2A). Furthermore, a 1.0 mg/kg dose of CS extract significantly elevated ICSS thresholds while the same dose of nicotine alone did not, suggesting greater potency for CS extract in elevating ICSS thresholds. Effects of EC extract on ICSS did not differ from those of nicotine alone. CS extract, but not EC extract or nicotine alone, significantly inhibited total MAO activity measured *in vitro*. CS extract also exhibited greater potency at inhibiting MAO-B activity *in vitro* compared to MAO-A activity. None of the formulations inhibited MAO activity measured *ex vivo*.

The greater ICSS threshold-elevating effects of CS extract compared to nicotine alone suggests that it produces greater acute aversive/anhedonic effects and, hence, has lower abuse liability. To the extent that our findings are predictive of tobacco use in humans, they indicate that the greater abuse liability of cigarettes compared to ANDS reflects factors others than the centrally-mediated effects of MAO inhibitors and other non-nicotine constituents in CS (e.g., nicotine content/yield, sensory factors, marketing). However, confirmation of our findings using other approaches is needed. For example, while the current approach involving evaluation of acute drug exposure on ICSS is a reliable predictor of abuse potential (Wise, 1996; Negus and Miller, 2014), evaluating effects of chronic dosing of extracts and nicotine alone on ICSS would allow study of changes in tolerance to nicotine’s aversive effects and would better model the chronic nature of tobacco product use in humans. Comparison of the primary reinforcing effects of extracts and nicotine alone in an i.v. self-administration model is also needed to clarify the relevance of our findings to tobacco product consumption. Finally, given the considerable differences in constituent profiles between brands of cigarettes and ECs (e.g., Jain et al., 2019; Eshraghian and Al-Delaimy, 2021), evaluating the effects of extracts of other products is needed to better understand the generality of the current findings.

Further evaluation of the increased acute aversive/anhedonic effects of CS extract, including identification of the responsible constituent(s), could help inform FDA policy and product standards. For example, we have suggested that the ICSS threshold-elevating effects of high nicotine doses may be related to toxicity (LeSage et al., 2016; Harris et al., 2018b). As such, while the constituents accounting for the effects of CS extract would not be included on the FDA’s list of harmful or potentially harmful constituents (HPHCs) as addiction-related chemicals, they may still need to be considered for inclusion on the list as toxic compounds. Evaluating the role of neurobiological mechanisms implicated in nicotine aversion [e.g., activity of α5 nicotinic acetylcholine receptors in the habenula-interpeduncular pathway (Fowler et al., 2011; Fowler and Kenny, 2014; Wills et al., 2022)] in the effects of CS extract could also provide important insights into the basic mechanisms mediating tobacco addiction.

The current findings provide the most comprehensive chemical characterization of tobacco product extracts in a preclinical addiction study. The higher levels of most behaviorally active non-nicotine constituents in CS extract than in EC extract is consistent with prior analytical studies of CS and EC aerosol (e.g., Belushkin et al., 2020). The only exception was that PG levels were higher in EC extract, although this was expected given that PG is a primary ingredient in ECs, but not cigarettes (e.g., Margham et al., 2016). The rank-order prevalence of minor alkaloids, β-carbolines, and carbonyls in the current CS extract was generally similar to that reported for Marlboro Gold cigarette smoke in an analytical study conducted in the same laboratory (Jain et al., 2019). However, absolute levels of non-nicotine constituents (expressed as percent of nicotine) tended to be lower in our CS extract. This may reflect our use of a different solvent than those used for constituent extractions in the Jain et al. (2019) study, which are not safe for use in animals. These conclusions should be considered preliminary, however, until confirmed in a formal analytical study that contemporaneously compares the constituent profiles of extracts and cigarette smoke/EC aerosol itself using the same lot of tobacco products. Nonetheless, these findings support further use of extracts generated by the present methods to characterize determinants of the abuse liability of these or other tobacco products.

Several non-nicotine constituents found in CS extract could account for its greater aversive/anhedonic effects compared to nicotine alone. For example, we found that several β-carbolines (MAO inhibitors) (e.g., harmane, norharmane) and minor alkaloids (e.g., nornicotine, anabasine) elevated ICSS thresholds when administered alone (Harris et al., 2015a,^2020^). In addition, while their effects on ICSS thresholds have not been studied, the carbonyls acetaldehyde and formaldehyde can produce aversive effects in other assays (Weisinger et al., 1974; Brown et al., 1978). Although doses of these constituents that produced aversive effects in those studies were higher than those delivered in CS extract in this study, these and other constituents may have additive or synergistic effects that increase aversion when they are combined in CS extract. Future studies involving fractionation of extracts (e.g., Noya et al., 2013) or evaluation of cocktails of nicotine and one or more of these constituents are needed to isolate the constituent(s) accounting for our findings.

Effects of a drug on ICSS are often predictive of its abuse liability in an i.v. self-administration (SA) model (Negus and Miller, 2014). Our ICSS data therefore contrast with some studies reporting greater abuse liability for CS extract than nicotine alone on certain measures of i.v. SA (e.g., Costello et al., 2014; Brennan et al., 2015; Marusich et al., 2019). This discrepancy could reflect numerous differences between behavioral models including route of administration (s.c. versus i.v.), dosing regimen (acute versus chronic), and contingency of drug exposure (experimenter-administered versus self-administered). It should also be noted that the SA literature in this area is mixed, with some studies reporting *lower* levels of SA of CS extract than nicotine alone (Gellner et al., 2016; Levin et al., 2021). Factors that could contribute to these inconsistencies across SA studies include differences in tobacco products, extract preparation procedures (solvent, pH adjustment), training procedures (dose, prior food training), schedule of drug delivery, and form of response (nose-poke vs. lever press), among others. Our ICSS data also raise the possibility that findings from studies reporting lower levels of SA of CS extract than nicotine alone may have reflected greater aversive effects of CS extract. Direct comparison of i.v. SA of the current CS and EC extracts and nicotine alone is needed to assess the convergent validity of the present findings.

In contrast to our current findings with EC extract, we previously found that EC *liquids* for three other products had reduced ICSS threshold-elevating effects compared to nicotine alone (LeSage et al., 2016; Harris et al., 2018b), suggesting reduced aversive/anhedonic effects. PG itself also attenuated nicotine’s ICSS threshold-elevating effects, including at concentrations similar to those in the EC liquids (Harris et al., 2018a). A possible explanation for the current findings is that levels of PG in EC extract, while higher than in CS extract, were nonetheless considerably lower than in the EC liquids studied previously. For example, at a 1.0 mg/kg nicotine dose, EC extract contained ≈0.5% PG whereas the EC liquids contained ≈1.0–3.0% PG depending on the product. This difference in PG levels may reflect our use of an EC aerosol extract rather than an EC liquid and/or our use of a different product, as both of these factors could influence constituent levels (e.g., Eshraghian and Al-Delaimy, 2021). Regardless, the PG levels in EC extract may have been insufficient to attenuate the ICSS threshold-elevating effects of high nicotine doses. Other factors that could account for the discrepancy include differences in levels of constituents other than PG in Vuse EC extract versus the previously studied EC liquids, as well as differences in rat strains used across studies (Sprague Dawley vs. Holtzman).

Our finding that CS extract but not nicotine alone inhibited MAO activity *in vitro* is consistent with prior studies (e.g., Castagnoli et al., 2002; Lewis et al., 2012; Costello et al., 2014), and may be at least partially attributable to the effects of harmane and/or norharmane in CS extract (see Herraiz and Chaparro, 2005). These *in vitro* findings also complement reports of MAO-A and MAO-B inhibition in the brains of smokers (e.g., Fowler et al., 1996). The more potent inhibition of MAO-B than of MAO-A by CS extract contrasts with prior studies reporting more potent inhibition of MAO-A than MAO-B for other CS extracts (Costello et al., 2014; van der Toorn et al., 2019). One possible reason for this discrepancy is the use of different products across studies, as one study found that the relative inhibition of MAO-A versus MAO-B by CS extracts differed across brands of cigarettes (Lewis et al., 2012). Comparison of our findings to these prior studies is also complicated by differences in procedures for assaying MAO inhibition.

The lack of effects of Vuse Solo Original EC extract on MAO activity *in vitro* contrasts with a report that EC liquid of this same product inhibited MAO *in vitro* (Truman et al., 2019). However, the magnitude of that effect was among the weakest on the panel of EC liquids in that study. In addition, constituents accounting for the MAO inhibitory effects of Vuse EC liquid (potentially vanillin and ethyl vanillin, see Truman et al., 2019) may not have effectively transferred from EC liquid to EC aerosol during EC extract preparation. For example, ≈20% of vanillin can be lost during aerosolization of an EC liquid (Erythropel et al., 2019).

Our finding that CS extract inhibited MAO *in vitro* but not *ex vivo* is consistent with the findings of Costello et al. (2014). One explanation for this finding is that concentrations of CS extract that inhibited MAO *in vitro* in the current study (≥≈15 μM nicotine following dilution in the assay) were likely higher than peak brain nicotine levels produced following extract injection in the ICSS study [≤≈7.7 μM, based on Tuncok et al. (2001)]. However, such direct extrapolation of *in vitro* to *in vivo* data is often inappropriate (e.g., Yoon et al., 2012). It is also possible that CS extract produced only reversible MAO inhibition *in vivo*. In contrast to non-reversible MAO inhibition such as that produced by TCP, reversible MAO inhibition would likely be lost during brain homogenization for *ex vivo* analysis (see Costello et al., 2014; Smith et al., 2015). Administration of CS extract using a chronic dosing regimen that better simulates long-term smoking may also be required to inhibit MAO measured *ex vivo*. Use of other approaches (e.g., measurement of brain monoamine levels following CS extract exposure using *in vivo* microdialysis) is needed to clarify the potential involvement of MAO inhibition in our findings.

A potential concern is that our use of a saline/ETOH solvent for extract preparation may have influenced our findings due to the well-established abuse liability of ETOH and its potential effects on MAO activity (Truman et al., 2019), as well as the ability of ETOH to interact with nicotine on certain measures of abuse liability (e.g., Schaefer and Michael, 1992; Tizabi et al., 2007). We therefore studied whether the highest concentration of ETOH present in the extracts (32%) would itself affect ICSS or MAO activity when administered alone. In addition, to control for any interactions between nicotine and ETOH, the same ETOH concentrations present in the extract doses were added to the respective nicotine alone doses (e.g., 32% ETOH at 1.25 mg/kg nicotine, 24% at 1.0 mg/kg nicotine, etc.). The 32% concentration of ETOH had no effects on baseline ICSS when administered alone, and the current ICSS dose-effect function for nicotine alone delivered in a saline/ETOH vehicle was similar to that observed previously for nicotine alone delivered in only saline (e.g., LeSage et al., 2016; Harris et al., 2018b). ETOH also did not affect MAO activity when administered alone or in combination with nicotine alone or EC extract in Experiment 3. Given the small volumes used in the ICSS and MAO studies (≈0.3–0.4 ml and 20 μl, respectively) and the resulting ETOH doses or concentrations produced (≤≈250 mg/kg for ICSS and 1% following dilution in the MAO assay, respectively), the lack of effects of ETOH on ICSS or MAO activity when administered alone or in combination with nicotine is expected (Kornetsky et al., 1988; Schaefer and Michael, 1992; Truman et al., 2019) and suggest that our use of ETOH as a solvent was not a confound. Nonetheless, inclusion of a control condition of nicotine delivered in only saline in future studies would help clarify this issue further.

The MAO assay used in this study may represent a further limitation. We used this assay because it is commercially available and is widely used in the literature (e.g., Malin et al., 2013; Smith et al., 2015, 2016; Matthews et al., 2018), including for evaluating effects of tobacco constituents (e.g., harmane and norharmane) (Smith et al., 2015). Nonetheless, the selectivity and specificity of this assay can be compromised by the presence of interfering substances in the samples (e.g., phenolic compounds, see Herraiz et al., 2018). In addition, given that this assay involves detection of H_2_O_2_, any H_2_O_2_ present in the extracts could have artificially increased apparent MAO activity. This seems unlikely, however, given that CS extract reduced rather than increased MAO activity *in vitro*, while EC extract had no effect (see Figure 4B). Nonetheless, our findings should be confirmed using chromatographic assays that avoid limitations of the current MAO assay (see Herraiz et al., 2018).

Despite its limitations, our study suggests that the non-nicotine constituents in the present CS extract do not contribute to the greater abuse liability of cigarettes compared to ANDS apparent in humans, and may actually limit the abuse liability of cigarettes by enhancing their aversive effects at high nicotine doses. Studies using self-administration models are needed to examine how such aversive effects might influence the relative consumption of CS and ANDS extracts. Future studies of similarly-prepared extracts of these and other products could be useful to clarify further the role of MAO inhibition and other mechanisms in the addiction-related effects of CS extract and could help inform FDA regulation of tobacco products.

## Data Availability Statement

The original contributions presented in the study are included in the article/supplementary material, further inquiries can be directed to the corresponding author/s.

## Ethics Statement

The animal study was reviewed and approved by the Institutional Animal Care and Use Committee of the Hennepin Healthcare Research Institute.

## Author Contributions

ML and AH supervised the conduct of the study and were responsible for the conception and design of the study. PM assisted with developing specific protocols, daily conduct of the experiment, and compiling data. IS and AA prepared the extracts and conducted the constituent analyses. AH wrote the first draft of the manuscript. All authors contributed to and have approved the final manuscript.

## Author Disclaimer

The content is solely the responsibility of the authors and does not necessarily represent the official views of the National Institutes of Health or Food and Drug Administration.

## Conflict of Interest

The authors declare that the research was conducted in the absence of any commercial or financial relationships that could be construed as a potential conflict of interest.

## Publisher’s Note

All claims expressed in this article are solely those of the authors and do not necessarily represent those of their affiliated organizations, or those of the publisher, the editors and the reviewers. Any product that may be evaluated in this article, or claim that may be made by its manufacturer, is not guaranteed or endorsed by the publisher.
